# Identify clear cell renal cell carcinoma related genes by gene network

**DOI:** 10.18632/oncotarget.22769

**Published:** 2017-11-30

**Authors:** Fangrong Yan, Yue Wang, Chunhui Liu, Huiling Zhao, Liya Zhang, Xiaofan Lu, Chen Chen, Yaoyan Wang, Tao Lu, Fei Wang

**Affiliations:** ^1^ Research Center of Biostatistics and Computational Pharmacy, China Pharmaceutical University, Nanjing, P.R. China; ^2^ Zhongda Hospital Southeast University, Nanjing, P.R. China; ^3^ State Key Laboratory of Natural Medicine, China Pharmaceutical University, Nanjing, P.R. China

**Keywords:** clear cell renal cell carcinoma, gene network, gene marker, gene selection, gene expression

## Abstract

Clear cell renal cell carcinoma (ccRCC) is the most prominent type of kidney cancer in adults. The patients within metastatic ccRCC have a poor 5-year survival rate that is less than 10%. It is essential to identify ccRCC -related genes to help with the understanding of molecular mechanism of ccRCC. In this literature, we aim to identify genes related to ccRCC based on a gene network. We collected gene expression level data of ccRCC from the Cancer Genome Atlas (TCGA) for our analysis. We constructed a co-expression gene network as the first step of our study. Then, the network sparse boosting approach was performed to select the genes which are relevant to ccRCC. Results of our study show there are 15 genes selected from the all genes we collected. Among these genes, 7 of them have been demonstrated to play a key role in development and progression or in drug response of ccRCC. This finding offers clues of gene markers for the treatment of ccRCC.

## INTRODUCTION

Renal cell carcinoma (RCC) is eighth highest cause of cancer mortality in adults, counting for almost 3% of all human malignancies [1]. Clear cell RCC (ccRCC), the most common type of RCC, accounts for about 80% of RCC cases [2–4]. Most of ccRCC patients usually present initially with localized disease, treated with surgery. But unfortunately, approximately 30% of ccRCC patients with localized disease eventually develop metastases that leads to a poor 5-year survival rate that is less than 10% [5]. With the advent of advanced development of gene sequencing technology, many studies have focused on the molecular mechanism of cancers aimed to understand insight of cancers. As for ccRCC, there are evidences showing that some important genes play key roles in ccRCC tumor like frequent mutation or methylation of the tumor suppressor gene (VHL) [6], frequent mutations of PBRM1, BAP1, SETD2 and KDM5C genes [7–9].

In genomic cancer studies, gene network analysis is useful to help researchers to understand the biological function and development of cells and organisms. Gene network analysis can be informative sometimes because it can describe not only whether there is a connection between two genes but also the strength of the connection which is more accurately to present complex interactions like co-expression or regulatory connection between genes. Previous studies about ccRCC have focused on the differentially expression genes only which potentially serve role in the ccRCC [10, 11], or on identification of genes which express differentially associated with metastasis in ccRCC [12]. Although many studies about ccRCC have large of scale efforts, little of studies use gene network to reveal molecular mechanism of ccRCC.

Thus, in this literature, we aim to identify gene markers associated with ccRCC on the basis on constructing a gene co-expression network. First, we identify the differentially expressed genes between normal sample and ccRCC tumor samples. Subsequently, a gene co-expression network was constructed to reveal the behind biological functions among differentially genes. Genes related to ccRCC were identified using the Network Sparse Boosting approach [13]. The results of this study may help to understand the molecular mechanism of ccRCC and also offer potential markers for ccRCC treatment or drug development.

## RESULTS

### The differentially expressed genes

For our analysis, we did differential genes analysis first to reduce the dimension. 1691 genes from the initial 20532 genes which was collected form the TCGA. In these 1691 genes, 932 genes displayed up-regulated between normal samples and tumor samples, and 759 genes displayed down-regulated. To reduce noise, we removed genes whose expression estimates with counts in less than 20% of cases. 1675 genes were kept from this step.

### Gene co-expression network construction

**Figure 1:**
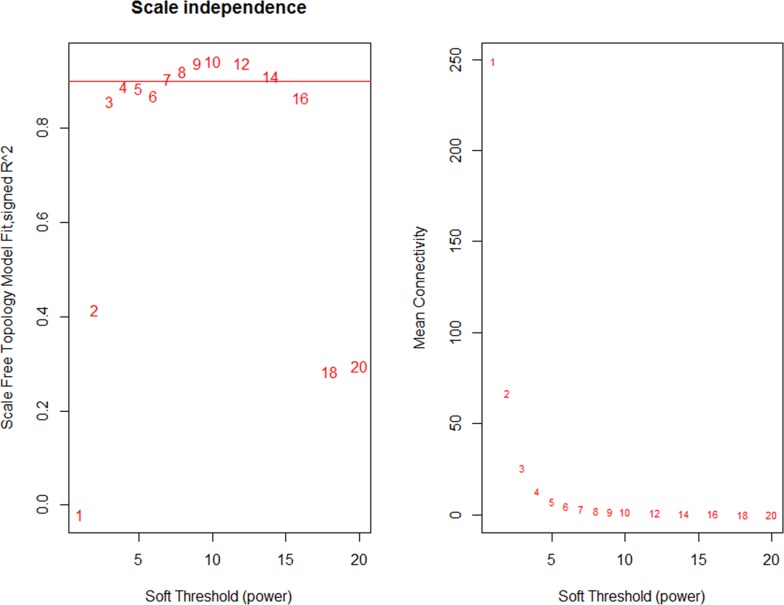
Analysis of network topology for various soft-thresholding powers The left panel shows the scale-free fit index (y-axis) as a function of the soft-thresholding power (x-axis). The right panel displays the mean connectivity (degree, y-axis) as a function of the soft-thresholding power (x-axis).

The 1675 node (genes) were used to construct a weighted gene co-expression network (WGCNA). To define the adjacency matrix *A* in the network, we need to determine the value of *β* to transfer similarity matrix into adjacency matrix. Figure 1 shows the trend of *β* value, according to WGCNA, we chose 7 as the soft threshold, which is the lowest power for which the scale-free topology fit index curve flattens out upon reaching a high value (in our data set, roughly 0.904).

**Figure 2:**
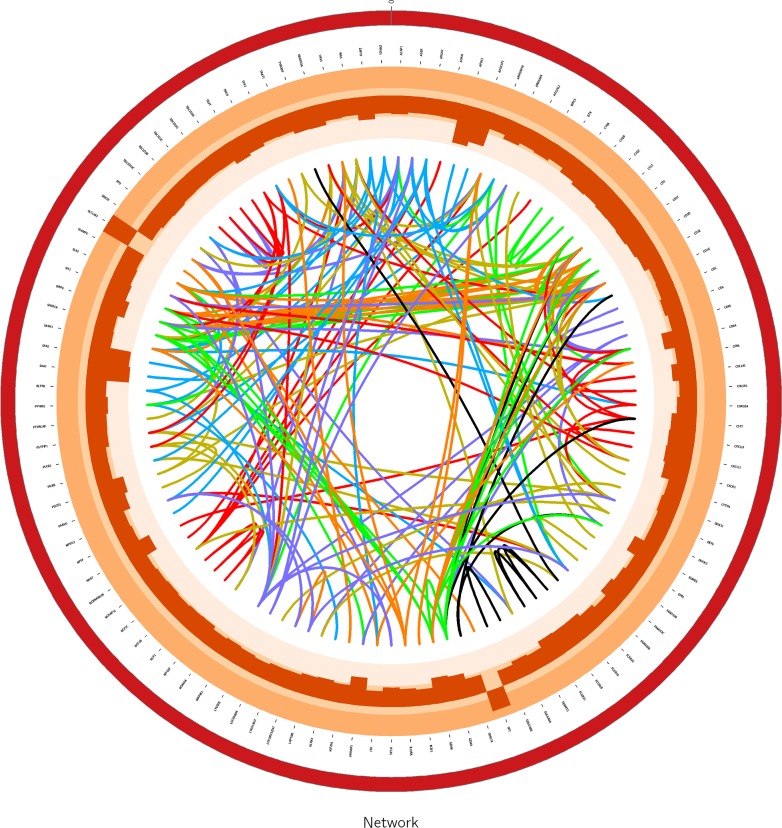
The graph for module4 in gene network using Circos software The links in center of the graph are edges which is greater than 0.5 between genes in the network. The histogram in the circle are the log-foldchange values of differentially expressed genes in network.

According to WGCNA, genes were finally divided into multiple modules (subnetworks) and genes in the same module may have similar biological functions. All 1675 genes were divided into 16 modules using WGCNA approach. Each module was represented by one color where the gray module which contained 71 genes was a noise module which was ignored. Thus, we kept the 15 modules except the grey module for our further analysis. To visualize the gene co-expression network, we used Circos software (http://circos.ca) to display the network (Figure 2).

### Gene functional annotation and Gene Ontology (GO) enrichment analysis for 15 modules

Gene functional annotation and Gene Ontology (GO) enrichment analysis for genes in 15 modules identified above discovered the behind biological function of each module by using DAVID 6.7 online tool. According to the results of GO enrichment analysis, each module related to different biological functions.

**Figure 3:**
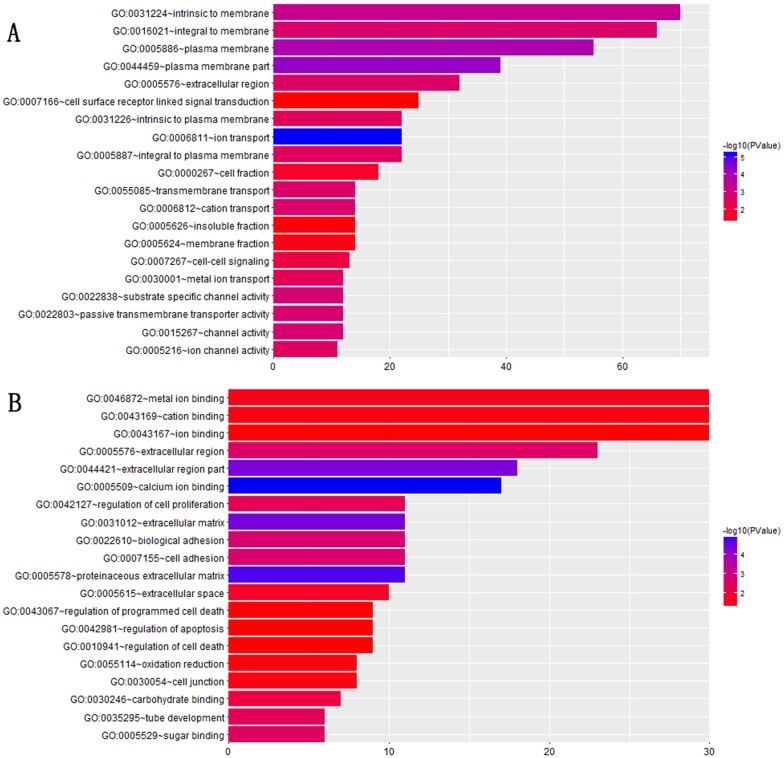
GO annotation and enrichment plot for **(A)** module1, **(B)** module2. The colors of each annotation depict the statistical significance of functional enrichment and the bars show the number of target genes contained in the corresponding annotation.

For instance, module1 which had 215 genes, a significant number of these 215 genes were related with the cellar component, (e.g. GO:0031224∼intrinsic to membrane, p-value=4.54E-04, 31.58% (66/215) GO:0016021∼integral to membrane, p-value= 0.002, GO: 0005886∼plasma membrane, p-value=1.34E-04, GO:0044459∼plasma membrane part, p-value=5.08E-05, GO:0005887∼integral to plasma membrane, p-value=0.003, GO:0031226∼intrinsic to plasma membrane, p-value=0.004, GO:0005576∼extracellular region, p-value=0.002), transport function, (e.g. GO:0006811∼ion transport, p-value=6.02E-06, GO:0006812∼cation transport, p-value =0.0002, GO:0055085∼transmembrane transport, p-value=0.0002, GO:0022803∼passive transmembrane transporter activity, p-value=0.00015, GO:0030001∼metal ion transport, p-value=0.0036), cell signal,(e.g. GO:0007267∼cell-cell signaling, p-value= 0.0089, GO:0007166∼cell surface receptor linked signal transduction, p-value=0.04), channel activity (e.g. GO:0015267∼channel activity, p-value=0.001, GO:0022838∼substrate specific channel activity, p-value= 0.001, GO:0005216∼ion channel activity, p-value= 0.003). For module2, containing 107 genes, a part of these genes were related to ion binding (e.g. GO:0046872∼metal ion binding, p-value= 0.032, GO:0043169∼cation binding, p-value= 0.037, GO:0043167∼ion binding, p-value= 0.044, GO:0005509∼calcium ion binding, p-value= 1.27E-05), cell process (e.g. GO:0042127∼regulation of cell proliferation, p-value=0.005, GO:0042981∼regulation of apoptosis, p-value=0.044, GO:0043067∼regulation of programmed cell death, p-value=0.046, GO:0010941∼regulation of cell death, p-value= 0.047), and extracellular region(e.g. GO:0005576∼extracellular region, p-value=0.0002, GO:0044421∼extracellular region part, p-value= 4.43E-05, GO:0005578∼proteinaceous extracellular matrix, p-value= 2.09E-05, GO:0031012∼extracellular matrix, p-value= 3.96E-05). Functional enrichment information for the two modules are visualized with bar graphs (Figure 3). The results of GO enrichment analysis for the rest of modules were displayed in Supplementary Materials (Supplementary Figure 1).

### ccRCC-related gene selection based on network

In order to define the ccRCC-related genes, we applied the NSBoosting approach to define genes which is related to ccRCC based on the network. According to the NSBoosting algorithm, 500 iterations were performed for each module in each step. 15 genes were selected eventually including LOC150197, SUSD4, HLA-G, C4orf49, LOC338588, CYS1, COL5A1, PLAU, GDNF, OTOA, IGFN1, C2orf40 (also known as MGARP), BARX2, HOXB13, MUC12. The differentially expressed results of 15 genes and the estimates of genes in NSBoosting were showed in Table 1. The iteration process of NSBoosting approach were displayed in Supplementary Materials (Supplementary Figures 2 and 3).

**Table 1:** The differentially expressed results and estimates of 15 selected genes using NSBoosting approach

Gene	Description	Padj^a^	Log-foldchange^b^	Dysregulation form^c^	Estimates^d^	Pre-reported^e^
**LOC150197**	long intergenic non-protein coding RNA 896	1.96E-02	3.6729	up	-0.1537	
**SUSD4**	sushi domain containing 4	1.73E-49	-3.7160	down	-0.1910	
**HLA-G**	major histocompatibility complex, class I, G	1.20E-20	2.5233	up	0.1558	√
**MGARP**	mitochondria localized glutamic acid rich protein	3.56E-07	2.8779	up	0.1602	
**SKA3**	spindle and kinetochore associated complex subunit 3	1.12E-02	2.0727	up	-0.2614	
**CYS1**	cystin 1	3.51E-67	-2.0583	down	0.1817	
**COL5A1**	collagen type V alpha 1 chain	4.17E-05	2.2068	up	0.2543	√
**PLAU**	plasminogen activator, urokinase	4.21E-27	-2.1804	down	-0.2805	√
**GDNF**	glial cell derived neurotrophic factor	2.70E-02	-2.0430	down	-0.2122	√
**OTOA**	otoancorin	4.75E-03	2.6513	up	0.3545	√
**IGFN1**	immunoglobulin-like and fibronectin type III domain containing 1	4.33E-02	5.6544	up	-0.1253	
**C2orf40**	chromosome 2 open reading frame 40	6.17E-08	-2.2598	down	0.1457	√
**BARX2**	BARX homeobox 2	4.19E-42	2.5958	up	0.2343	
**HOXB13**	homeobox B13	1.91E-02	3.5208	up	-0.1159	√
**MUC12**	mucin 12, cell surface associated	5.97E-05	4.0677	up	-0.1518	

^a^ Adjusted p-value is calculated in differential expression analysis with threshold of 0.05.

^b^ Log-foldchange is calculated in differential expression analysis with threshold of 2.

^c^ Dysregulation form indicates whether the corresponding gene is up- or down-regulated.

^d^ Estimates of selected genes is calculated in NSBoosting approach.

^e^ That a gene is pre-reported means some ccRCC-relevant research has been done before.

We searched on PubMed (https://www.ncbi.nlm.nih.gov/pubmed) to ensure that 15 genes were meaningful for ccRCC. Interestingly, 7 genes including HLA-G, COL5A1, PLAU, GDNF, OTOA, HOXB13 and C2orf40 were related with ccRCC in many ways like drug response, poor prognosis and so on. This results may make sure that the selection approach reasonable. There are still some genes’ function are not clear in tumors which means the functions of these gene in ccRCC should be further verified.

### Association between 15 selected gene expression levels and ccRCC prognosis

**Figure 4:**
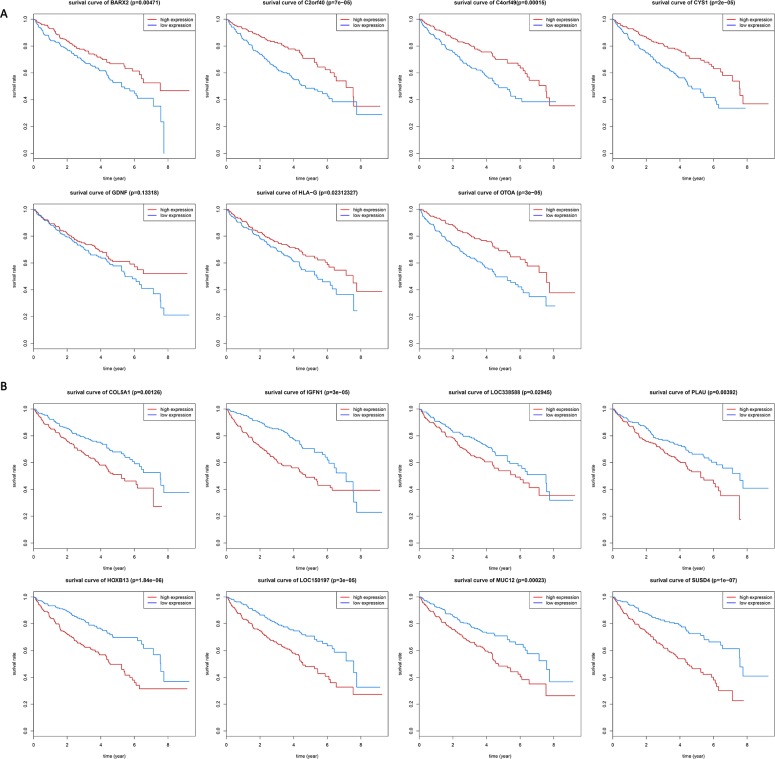
Kaplan-Meier (KM) survival curves for **(A)** 7 genes and **(B)** 8 genes. KM survival curves show significant overall survival differences between higher-expression levels and lower-expression levels of ccRCC patients.

To reveal association between 15 selected genes expression levels and ccRCC prognosis, we performed survival analysis. Association between 15 genes expression levels and ccRCC prognosis are significantly (log-rank test, P-value <0.05). Kaplan-Meier survival curves (Figure 4A) show that patients with lower expression levels of 7 genes including BARX2, C2orf40, C4orf49, CYS1, GDNF, HLA-G and OTOA have better overall survival prognoses than those with higher expression levels of these 7 genes in ccRCC. Patients with higher expression levels of the rest 8 genes (COL5A1, HOXB13, IGFN1, LOC150197, LOC338588, MUC12, PLAU and SUSD4) have significantly worse overall survival prognoses than those with lower expression levels of 8 genes (Figure 4B). These results indicated that all these 15 genes are frequently associated with poor clinical outcomes in ccRCC.

## DISCUSSION

In this study, we aim to identify ccRCC-related genes according raw sequencing data from TCGA based on the gene network effect. The construction of gene network provides an insight of correlation between genes and reveals the complex biological functions. The first step of our study is constructing a gene network. We adopt the weighted co-expression gene network to describe the correlation between genes. With WGCNA, genes are divided into multiple modules which means genes in the same modules tend to have a similar biological functions. The second step is selecting the key gene related to ccRCC based on the previously constructed network. We chose AFT model as the basic statistics model combing the NSBoost approach to identify the ccRCC-related genes.

There are some advantages of the approached we applied. We consider the complex relationships between genes to improve the accuracy of gene selection. Thus, we constructed a gene network to represent the relationship between genes. On the other hand, the selection approach is based on gene network which could make the results more biological meaningful.

The finally results is reasonable. 7 genes of the selected 15 genes, has been reported in many studies to be associated with ccRCC in different ways. For instance, Gene HLA-G has been reported before to reveal its expression, regulation, structure and function in renal cell carcinoma [14–16]. In addition, HLA-G-regulatory miRNAs like mir-548q and mir-628-5p were identified. The two overexpression miRNAs in ccRCC cell line caused a downregulation of HLA-G gene and protein and mir-548q could be able to revert to the immune escape of HLA-G expression tumor cells [17]. Gene C2orf40, also known as ECRG4, was found that it is regulated by DNA methylation and its downregulation in ccRCC is associated with poor prognosis [18], which means ECRG4 may be a marker for ccRCC survival. Aberrant promoter hypermethylation is a common mechanism for inactivation of tumor suppressor genes in cancer cells. Gene PLAU, plasminogen activator, urokinase, was showed that cancer cell-specific methylation in RCC cell lines [19]. Gene COL5A1, has shown that its deregulated level was caused by mir-25-3p in renal cancer. This may influencing cancerous adhesion [20]. Moreover, Okuda and co-authors [21] found that the methylation status of HOXB13 correlated with the loss of its expression both in RCC lines and primary tumors, and methyltransferase inhibitor treatment induced the recovery of its expression. Exogenous expression of HOXB13 in RCC cells that lacked endogenous HOXB13 expression suppressed colony formation and induced apoptotic features. Furthermore, HOXB13 methylation correlated positively with tumor grade and microvessel invasion. These results suggest that HOXB13 is a novel candidate tumor suppressor gene in RCC and that its inactivation may play an important role in both RCC tumorigenesis and progression. As for drug response, gene GDNF is associated with cellular targets of sorafenib, the first oral multikinase inhibitor that targets Raf and affects tumor signaling and the tumor vasculature [22]. The rest part of 15 genes have not been reported to relevant with ccRCC yet, but need to be further validated to play a role in renal clear cell carcinoma.

## MATERIALS AND METHODS

### Materials

We collected the mRNA-Seq gene expression data (Level 3) and clinical data for the Kidney Renal Cell Carcinoma (KIRC) form the Cancer Genome Atlas. Both the two types data were download form Firehose (http://firebrowse.org). For mRNA-Seq data, raw counts workflow type was used with 537 samples of mRNA expression data. For clinical data, 537 sample with sufficient clinical information were used. Before our analysis, data processing was done first as follows:

First, the normal sample (68 cases in mRNA set) and tumor samples (469 cases) were identified. Second, the tumor samples were matched among mRNA data and clinical data. A data set that consists of a total 469 tumor samples with these two types data was available.

### Detection of differentially expressed genes

We identified differentially expressed genes between normal samples and tumor samples for KIRC first for our analysis with Deseq R package [23]. We used the threshold of adjusted p-value <0.05 and log-foldchange >2 to identify the differentially expressed genes.

### Gene expression data normalization

As read counts follow a negative binomial distribution, which has a mathematical theory less tractable than that of the normal distribution, RNA-seq data was normalized with the voom methodology [24]. The voom method estimates the mean-variance of the log-counts and generates a precision weight for each observation. This way, a comparative analysis can be performed with all bioinformatic workflows originally developed for microarray analyses.

### Gene co-expression network construction

There are multiple ways to construct gene networks. In this study, we used the WGCNA approach [25] to construct a biological meaningful gene network. Many studies have constructed gene co-expression network using WGCNA approach like Giulietti *et al*, 2016; Sundarrajan *et al*, 2016 [26, 27]. The WGCNA approach is built on the understanding that the coordinated co-expression of genes encode interacting proteins with closely related biological function and cellular processes. According to the WGCNA, genes which have similar functions will be grouped in a module. The hub genes in a module, which are “well connected” with other a lot of genes, may be have important biological functions. Different modules in the network tend to have different biological functions. The algorithm of WGCNA was implemented by R package WGCNA [28] to construct the weighted co-expression network.

### Gene functional annotation and Gene Ontology (GO) enrichment analysis

Gene-annotation enrichment analysis with functional annotation clustering was performed for genes in each module that was discovered by WGCNA above by using DAVID 6.7 (https://david-d.ncifcrf.gov) to reveal the biological functions of each modules. DAVID 6.7 provides a comprehensive set of functional annotation tool for users to understand the biological meaning for a large list of genes.

### Basic statistical model for ccRCC-related gene selection

We used the basic accelerated failure time (AFT) model [29] for survival analysis using gene expression and survival data. In order to deal with censored data, weighted least squares method [30] was used for the AFT model rather than ordinary least squares (OLS) method, because weights are used to account for censoring in the least square criterion.

### ccRCC-related gene selection

In order to select more accurate and meaningful ccRCC-related genes, we applied Network Sparse Boosting (NSBoost) approach [11], the family of boosting approaches, which could consider the effect of gene network on ccRCC. The NSBoost is a variable selection approach which has a better interpretability than usual dimension reduction approached like Lasso because of its lower computational cost. We first explain the rationale of this method.

With NSBoost, ccRCC-related gene selection was achieved in two main steps based on gene co-expression network. In the network construction part, all genes were divided into multiple modules that do not overlap each other. In the first step, we analyzed each module respectively. According to the spirit of WGCNA, genes in the same module tend to have similar biological functions. Thus, it is sensible to analysis each module separately. For a specific module, we not only selected a group genes which are related to ccRCC with NSBoost but also constructed a *super marker* which is a linear combination of selected genes and can represent effects of all genes in this module. In the second step, joint effects of all super makers are be considered. It is necessary to conduct the second step of selection and discriminate ccRCC-associated modules from noises. With the above two steps, we identified which modules are related to ccRCC as well as which genes are ccRCC-related in the selected modules.

### Survival analysis

We performed survival analysis of ccRCC patients based on 15-gene expression data. Kaplan-Meier survival curves were used to show the overall survival differences between 15-gene higher-expression-level patients and 15-gene lower-expression-level patients. Higher-expression-level and lower-expression-level patients were determined by the median values of 15 selected expression. If the gene expression level in a patient was higher than the median value, the patient was classified as higher-expression-level; otherwise as lower-expression-level. We used the log-rank test to calculate the significance of survival-time differences between two classes of patients with a threshold of P-value < 0.05.

## SUPPLEMENTARY MATERIALS FIGURES


