# Supplementary material for: Identify clear cell renal cell carcinoma related genes by gene network
Source: Oncotarget. 2017 Nov 30;8(66):110358–66. doi: 10.18632/oncotarget.22769 (PMC5746388; doi:10.18632/oncotarget.22769)
Supplement: Supplementary file 1 [file oncotarget-08-110358-s001.pdf]

Identify clear cell renal cell carcinoma related genes by gene network

SUPPLEMENTARY MATERIALS

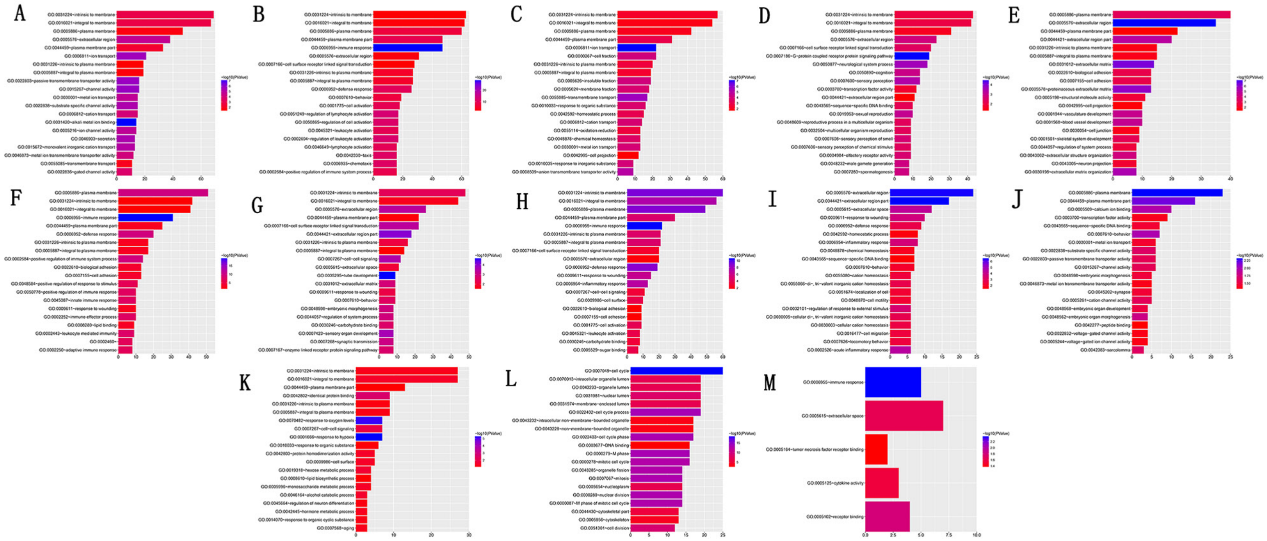

Supplementary Figure 1: GO annotation and enrichment plot for (A-M) module3 – module15. The colors of each annotation depict the statistical significance of functional enrichment and the bars show the number of target genes contained in the corresponding annotation.

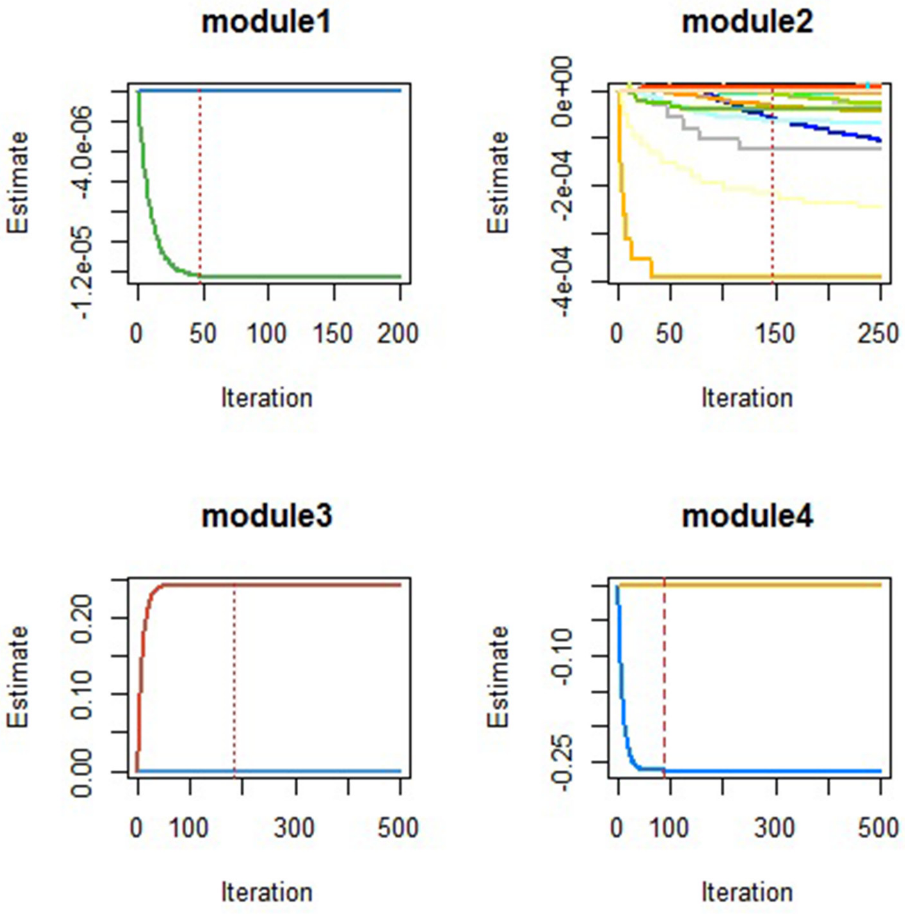

Supplementary Figure 2: The four panels correspond to four modules of 15 modules in Step 1 within NSBoost. The vertical dashed lines in each panels represent the corresponding stopping iterations.

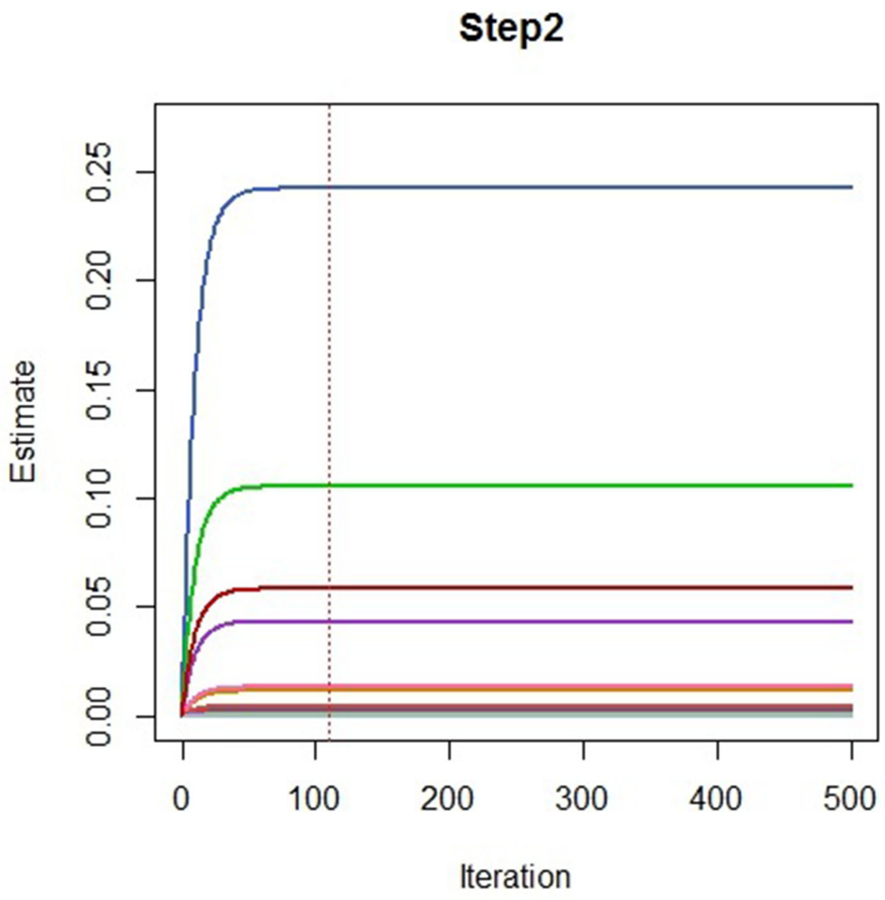

Supplementary Figure 3: The panel corresponds to15 super markers in Step 2 within NSBoost. The vertical dashed line in the panel represents the corresponding stopping iteration.
